# Optimizing NK-92 serial killers: gamma irradiation, CD95/Fas-ligation, and NK or LAK attack limit cytotoxic efficacy

**DOI:** 10.1186/s12967-022-03350-6

**Published:** 2022-04-02

**Authors:** Lydia Navarrete-Galvan, Michael Guglielmo, Judith Cruz Amaya, Julie Smith-Gagen, Vincent C. Lombardi, Rebecca Merica, Dorothy Hudig

**Affiliations:** 1 University of Nevada, Reno School of Medicine, Reno, NV 89557 USA; 2 Fred Hutchinson Cancer Research Center, Seattle, WA 98109 USA; 3 University of Nevada, Reno School of Community Health Sciences, Reno, NV 89557 USA; 4 Biology Department, St. Olaf College, Northfield, MN 55057 USA

**Keywords:** NK-92, NK, Radiation, Fas/CD95, Serial killing, Lymphokine activated killer, Limitations, Adoptive cell transfer, Therapeutic efficacy

## Abstract

**Background:**

The NK cell line NK-92 and its genetically modified variants are receiving attention as immunotherapies to treat a range of malignancies. However, since NK-92 cells are themselves tumors, they require irradiation prior to transfer and are potentially susceptible to attack by patients’ immune systems. Here, we investigated NK-92 cell-mediated serial killing for the effects of gamma-irradiation and ligation of the death receptor Fas (CD95), and NK-92 cell susceptibility to attack by activated primary blood NK cells.

**Methods:**

To evaluate serial killing, we used ^51^Cr-release assays with low NK-92 effector cell to target Raji, Daudi or K562 tumor cell (E:T) ratios to determine killing frequencies at 2-, 4-, 6-, and 8-h.

**Results:**

NK-92 cells were able to kill up to 14 Raji cells per NK-92 cell in 8 h. NK-92 cells retained high cytotoxic activity immediately after irradiation with 10 Gy but the cells surviving irradiation lost > 50% activity 1 day after irradiation. Despite high expression of CD95, NK-92 cells maintained their viability following overnight Fas/CD95-ligation but lost some cytotoxic activity. However, 1 day after irradiation, NK-92 cells were more susceptible to Fas ligation, resulting in decreased cytotoxic activity of the cells surviving irradiation. Irradiated NK-92 cells were also susceptible to killing by both unstimulated and IL-2 activated primary NK cells (LAK). In contrast, non-irradiated NK-92 cells were more resistant to attack by NK and LAK cells.

**Conclusions:**

Irradiation is deleterious to both the survival and cytotoxicity mediated by NK-92 cells and renders the NK-92 cells susceptible to Fas-initiated death and death initiated by primary blood NK cells. Therefore, replacement of irradiation as an antiproliferative pretreatment and genetic deletion of Fas and/or NK activation ligands from adoptively transferred cell lines are indicated as new approaches to increase therapeutic efficacy.

**Supplementary Information:**

The online version contains supplementary material available at 10.1186/s12967-022-03350-6.

## Background

Natural killer (NK) cells are appealing cells for immunotherapy because they are very potent effector lymphocytes of the innate immune system that can attack and kill many different tumor target cells without prior sensitization [1–3]. Studies show that some cytotoxic immune cells, including T- and NK cells, are capable of killing multiple target cells, sequentially, in a process termed serial killing [4, 5]. Moreover, serial killer cells are often faster at delivering lytic hits and inducing target cell death than non-serial killer NK cells [6]. Unfortunately for adoptive immunotherapies, many NK cells kill only once, with less than 30% capable of serial killing [7–9]. While the number of target cells killed per effector varies by study, it is consistently noted that a minority of NK cells is responsible for the majority of killing events. Furthermore, there are challenges to obtaining sufficient numbers of functionally active NK cells from a patient’s blood because, despite being widespread throughout the body, NK cells represent just 2–18% of lymphocytes in human peripheral blood and it is challenging to obtain sufficient numbers of NK cells needed to overwhelm the number of tumor cells [10, 11].

To readily obtain the large numbers of NK cells needed, immortalized cytotoxic cell lines have been established from patients with NK-cell cancers; however, little is known about their serial killing capacities. NK-92 are one of over eight available NK cell lines (Additional File 1: Table S1) and have reproducible cytotoxicity to a variety of tumor types [10, 12, 13], even under hypoxic conditions [14]. As of February 2022, NK-92 cells are the only NK cell line approved by the FDA for clinical trials and have been the subject of over 550 publications cited in PubMed USA. In addition to their innate activities, NK-92 cells can also be genetically manipulated to express receptors that recognize specific tumor antigens and that augment therapeutic monoclonal antibodies through antibody-dependent cellular cytotoxicity (ADCC). These NK-92 cells and CAR-NK-92 variants are immediately available and more affordable than current CAR-T-cell therapy [15]. In fact, haNK (NK-92) cells, engineered to express the high affinity CD16A allele (in order to recognize tumor cell-bound monoclonal antibodies), were tested in combination with anti-PD-L1 antibody, avelumab, and have now been further modified to also express a PD-L1-specific chimeric antigen receptor [16, 17]. NK-92 cells have been infused into patients with advanced cancers, resulting in clinical benefits with limited side effects. Additionally, NK-92 cells are being tested in several clinical trials in four different countries and for patients with a range of malignancies, including leukemia, glioblastoma, and melanoma [18–21]. It is important to assess what can happen to these cells in vivo, following transfer, a challenging issue that has been addressed so far only by monitoring cells circulating in the patients’ blood [19]. In this report, we assessed in vitro*,* potential hazards to NK-92 cell serial killing that could occur in vivo after adoptive transfer, including losses of cytotoxic serial capacity following irradiation, ligation of NK-92 cell Fas by cells residing in the tumor, as well as vulnerability of the NK-92 cells to attack by blood primary NK cells.

As far as the authors are aware, we are the first to observe killing frequencies [5] (KF) > 1 by NK-92 cells using standard release assays (presented at American Association of Immunologists annual meeting, 2021). We monitored this serial killing to predict potential losses of activity to the cells during the time that the cells remain viable after adoptive transfer. Our data demonstrate several potential complications that would result in losses to therapeutic efficacy in vivo when NK-92 cells become impaired following irradiation. It was previously reported that when NK-92 cells were irradiated with 10 Gy, NK-92 cell proliferation was prevented and cytolytic activity was substantially conserved within the live cells remaining 1 day following irradiation [22]. We found, however, that NK-92 cell serial killing significantly decreased 1 day after irradiation. Irradiation also increased NK-92 cell susceptibility to Fas-ligation as well as to attack by lymphokine-activatable primary blood NK cells.

## Methods

### Cell lines and culture

All cell lines regularly tested negative for mycoplasma using the MycoAlert™ mycoplasma detection kit (Lonza, Walkersville MD). ***NK-92*** cells (ATCC CRL-2407) were cultured in alpha Minimum Essential Media with l-glutamine and sodium pyruvate, no ribonucleosides or deoxyribonucleosides (Gibco, Waltham MA), with 0.2 mM inositol, 0.2 mM 2-mercaptoethanol, and 0.02 mM folic acid, 12.5% horse serum (Gibco), 12.5% fetal bovine serum (FBS) (Atlanta Biologicals, Flowery Branch GA), and 1000 U/ml Tecin, Teceleukin recombinant interleukin 2 (IL-2) (Roche, Basel Switzerland) at 5% CO_2_ and 37 °C. ***K562*** cells (ATCC CCL-243) were cultured in Dulbecco’s modified Eagle Medium (DMEM) with 4.5 g/L glucose, l-glutamine, and sodium pyruvate (Corning Life Sciences) and with 10% fetal bovine serum (FBS) and 1% penicillin–streptomycin (pen-strep) solution (MilliporeSigma, Burlington MA) at 5% CO_2_ and 37 °C. ***Raji*** (ATCC CCL-86), ***Daudi*** (ATCC CCL-213), and ***Jurkat*** (ATCC TIB-152) cells were cultured in Roswell Park Memorial Institute (RPMI) media with l-glutamine (Corning Life Sciences, Tewksbury MA), 10% FBS and 1% pen-strep at 5% CO_2_ and 37 °C. ***PBMCs*** (STEMCELL Technologies, Vancouver, Canada) were either resting, unstimulated after culture in DMEM with 4.5 g/L glucose, l-glutamine, and sodium pyruvate and with 10% FBS and 1% Pen-Strep at 37 °C and 5% CO_2_ overnight or LAK activated, cultured in DMEM with 4.5 g/L glucose, l-glutamine, and sodium pyruvate and with 10% FBS and 1% pen-strep at 37 °C and 5% CO_2_ and supplemented with 1000 U/ml IL-2 for 3 days prior to assay.

### Cell irradiation

NK-92 cells were gamma irradiated in 15 ml of culture media, using a cesium^137^ source (JL Shepherd Model I-30). A dose range of 2.5–20 Gy was tested. After radiation, cells were cultured for the times indicated (0–48 h) in 1000 U/ml IL-2. Control cells were processed in parallel without applying irradiation.

### Fas-receptor ligation

*For cytotoxicity assays*, NK-92 cells were brought to 2.5 × 10^5^ cells/ml and cultured overnight with either 1 ug/ml LEAF purified anti-human CD95 clone EOS9.1 or LEAF purified mouse IgM clone MM-30 as an isotype control. *For flow cytometric assays of potential cell death induced after ligation of cellular Fas/CD95*, NK-92 or Jurkat cells were cultured overnight at 2.0 × 10^6^ cells/ml with 1 ug/ml LEAF purified anti-human CD95 or LEAF purified mouse IgM isotype control. When combined with irradiation, cells were first irradiated and then mAbs were added either immediately after irradiation or 1 day after irradiation, as indicated in the results. Control cells were processed in parallel without addition of antibodies. The mAbs were from BioLegend (San Diego, CA).

### Cytotoxicity assays

Target cells were labeled with Na^51^CrO_4_ (Perkin Elmer, Waltham, MA) [23]. Effector NK-92 cell counts were determined using a hemacytometer with routine samples of > 600 cells that excluded Trypan blue (MilliporeSigma). NK-92 effector cells were diluted twofold in quadruplicate replicas for each E:T in V-bottom plates (Costar 3894, 96 well) in 0.1 ml to create six to eight effector to target cell (E:T) ratios. NK cell effectors within PBMCs were diluted similarly. Radiolabeled ‘target’ cells (1 × 10^5^/ml in 0.1 ml) were added to each well to induce cytotoxicity. Plates were centrifuged (Sorvall RC 6 +) at 1000 rpm for 3 min to bring the effector and target cells together and incubated at 5% CO_2_ and 37 °C for 8 h (unless otherwise noted). After incubation, plates were centrifuged at 1200 rpm for 10 min and 0.1 ml of cell-free supernatant was removed for analysis in a Perkin-Elmer Wizard gamma counter. Spontaneous release was calculated using the average leak rate of target cells without effectors and the maximum release was the radioactivity released by target cells lysed with 1% SDS. The calculated % specific release is a measure of target cell killing, as targets release internalized ^51^Cr into the sampled supernatant when they die. Percent specific release was calculated using the following formula:

$$\% {\text{Specific Release }} = \, [\left( {Experimental \, counts \, {-} \, Spontaneous \, Release} \right)/(Max \, {-} \, Spontaneous \, Release)] \times { 1}00.$$

The data illustrated are representative of a minimum of three replicate experiments.

### Flow cytometry analysis

*For determining the presence of Fas-receptor CD95 on cells*, the AF647 anti-CD95 clone DX2 was used with Zombie Aqua to eliminate dead cells from consideration. Cells were taken immediately from culture, washed once with PBS, and stained with a 1:100 dilution of Zombie Aqua for 30 min at room temperature (RT), protected from light. Then cells were quenched with FACS buffer with 1% FCS and brought to 5 × 10^6^ cells/ml, aliquoted into flow tubes, stained with 10 ug/ml AF647 anti-CD95 clone DX2 for 30 min at RT, protected from light and washed twice. *For analysis of cell death following irradiation and/or anti-Fas ligation*, the following fluorescent probes were used: FITC annexin V (BioLegend, San Diego CA) and 7-aminoactinomycin D (7-AAD) (MilliporeSigma). Irradiated or anti-Fas treated, and control cells were washed with annexin V binding buffer and stained with 4 ug/ml 7-AAD and 4.5 ug/ml FITC annexin V for 20 min at RT, protected from light, then washed twice and brought up with annexin V buffer containing 20 ug/ml actinomycin D (AD) (MilliporeSigma), and fixed with 0.5% formaldehyde. *For determining NK counts within PBMCs*, TruCOUNT™ Beads (BD Biosciences) and the following fluorescent antibody panel was used: PacBlue anti-CD45 clone HI30, BV711 anti-CD56 clone HCD56, BV711 anti-CD16A clone 3G8, FITC anti-CD3 clone OKT3, AF647 anti-CD244 [24] clone C1.7, together with 7-AAD to identify necrotic cells, *without washing the cells* (in order to prevent cell loss). Gating sequence available in Additional file 2: Fig. S1. Resting or LAK activated PBMCs (as previously described) were taken from culture, spun down, and resuspended in FACS buffer, then aliquoted into flow tubes. Cells were stained for 30 min at RT, protected from light. After staining 20 ug/ml AD was added and the cells fixed by addition of formaldehyde (MilliporeSigma, Boston MA) to a final concentration of 0.5%. All mAbs were from BioLegend, San Diego CA, and titrated for the concentrations suitable for no-wash conditions. The samples were analyzed within 1 day, using a BD Biosciences Special Order Research Product LSR II analytical flow cytometer with a high throughput sampler (HTS) unit. *Cytometric data* were analyzed with FlowJo software (BD Biosciences) to determine cell counts, %positive cells, median fluorescence intensity (MFI), and statistical comparisons between samples. The data illustrated are representative of a minimum of two replicate experiments.

### Statistical analyses

*Cytotoxicity assay data* were calculated and graphed with Microsoft Excel and evaluated using SPSS Statistics (IBM, *version 28*, Armonk, NY) using linear regression analysis or difference-in-difference comparisons. LU_50_’s (the number of effector cells needed to cause 50% lysis) were calculated by linear regression equations of cytotoxicity (y = % specific ^51^Cr release, x = log_10_ of the E:T cell ratios) to determine the number of cells needed to kill 50% of the ‘target’ cells. Then the lytic activity was expressed as LU_50_/1.0 × 10^6^ effector cells [25, 26]. The slopes of this linear regression of cytotoxicity provide information that is useful to detect differences in cellular cooperativity or multiple ‘hits’ needed to kill ‘target’ cells. FlowJo “compare population” tool was used to calculate Overton subtraction [27] and chi-squared statistics to analyze flow cytometric populations.

## Results

### NK-92 cells serially kill multiple cancer cell lines

**Fig. 1:**
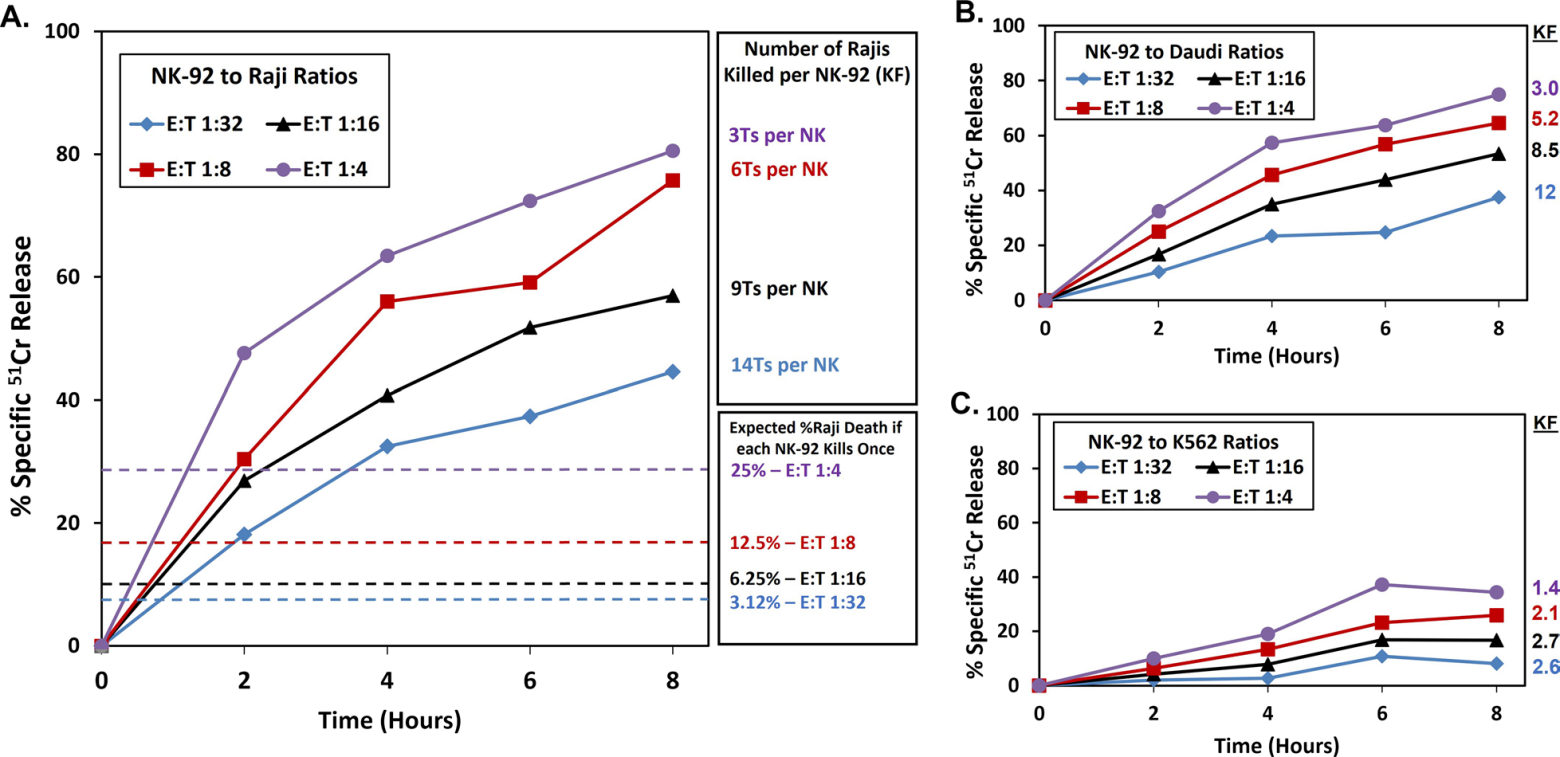
NK-92 cell cytotoxicity and serial killing frequencies of Raji, Daudi and K562 targets. Each colored line/symbol represents %specific release at a different E:T. Dashed lines represent the expected % dead targets if one target was killed by one effector at an E:T. **A** Raji cells as ‘targets’. **B** Daudi cells as ‘targets’. **C** K562 cells as ‘targets’. The three targets were assayed concurrently. The standard deviations for each data point were less than 2% specific ^51^Cr release. The cytotoxicity towards Raji cells is significantly greater than Daudi and K562 cells (E:T 1:32 linear regression p < 0.01 & p < 0.001, respectively)

Many tumor cells are suitable targets for serial killing because NK cells recognize a variety of ligands [1] that are on different tumors. The commonly used NK target cell, K562, was compared with two B-cell tumor lines, Daudi and Raji, because NK-92 cells were previously reported to kill these hematopoietic tumor cells well in vitro [28] and to kill hematopoietic tumors of patients in a clinical trial [21]. We used killer frequencies (KFs) to measure the average number of tumor ‘target’ cells killed per single NK-92 cell over time. The assays were stopped at 2-, 4-, 6-, and 8-h time points for measurement of cytotoxic activity. Raji cells were killed better than K562 and Daudi at all time-points. Looking specifically at the 8-h data and at an E:T of 1:16, Rajis have 57% specific release (KF = 9 dead per single NK-92 cell, calculated as 56% of targets killed divided by 6.25%, the frequency of effectors available at a 1:16 ratio) compared to 53% (KF = 8.5) and just 17% (KF = 2.7) for Daudi and K562 targets, respectively (Fig. 1A–C). Additionally, Raji and Daudi tumor cells continued to be serially killed after 6 h unlike K562 cells, where serial killing stopped after 6 h (Fig. 1B and C). For 8-h assays, at an E:T of 1:16, NK-92 cells had an average KF of 6.7 Raji per effector in 11 experiments, with a KF range of 3.0 – 9.5. NK-92 KFs increased as target concentrations increased, reaching a record of 14 Raji per NK-92 at an E:T of 1:32. The data in Fig. 1 represent the serial killing to each target that was observed within one experiment. Similar activities were observed within multiple experiments (Raji: n = 11 experiments, Daudi: n = 3, K562: n = 4).

Killing frequency (KF) describes the average number of targets killed per effector cell and assumes that every effector cell kills [5]. A single round of killing for each effector cell concentration is indicated by the corresponding, colored dashed lines in Fig. 1. It should be noted that in practice with NK-92 cells there is heterogeneity in killing. We observed variable externalization of CD107A associated with cytotoxic granule release [29] (Navarrete-Galvan et al., unpublished results) and variability in killing was also observed previously by time-lapse cinematography [30, 31]. Therefore, it is likely that an individual NK-92 cell can kill more targets than are reflected by the KF values. The KFs at E:Ts of 1:16 at 8 h and the number of experiments included in this study, with the means ± standard deviations for each target were: Raji, n = 11, KF 6.6 ± 2.2; Daudi, n = 3, KF 6.8 ± 1.6; and K562, n = 5, KF 1.6 ± 1.5. The KF ranges are in Additional file 3: Table S2.

### Irradiation of NK-92 cells impacts both their viability and their cytotoxic functionality

**Fig. 2:**
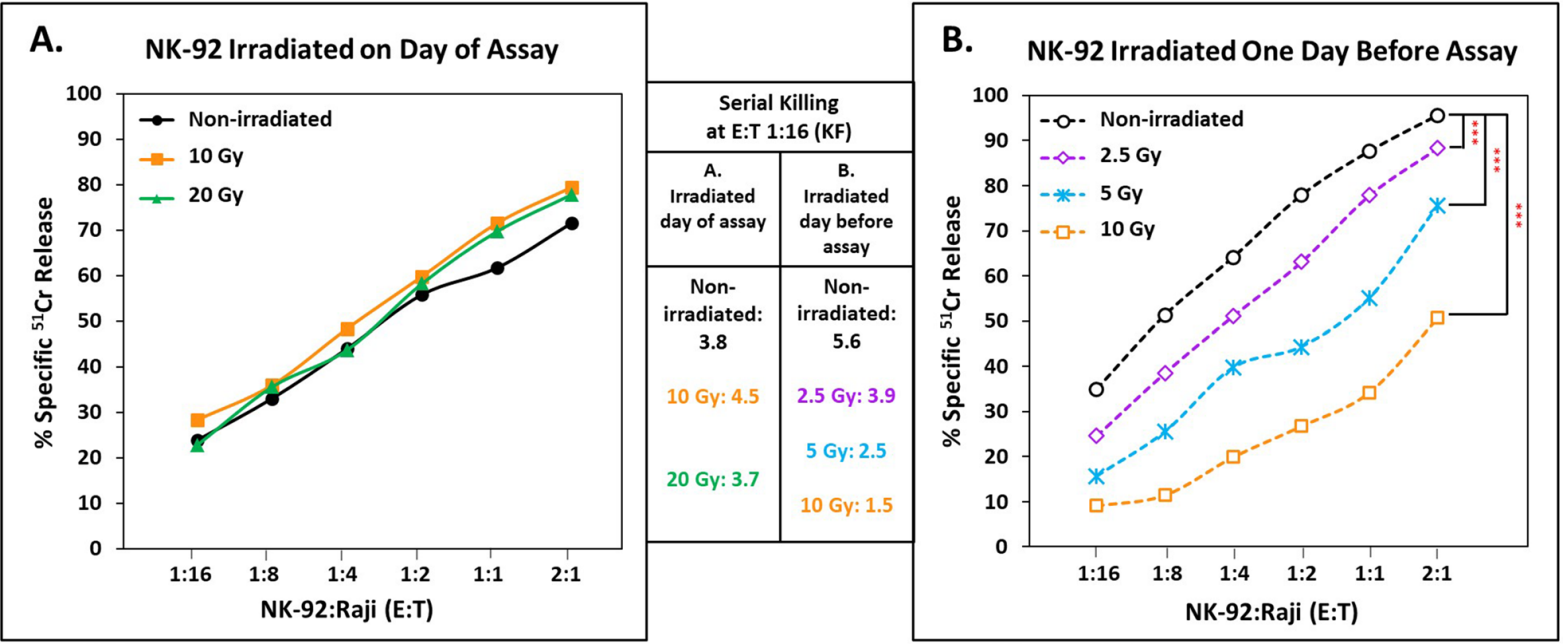
Effects of irradiation on serial killing. NK-92 cells were irradiated on **A** the day of experiment or **B** the day before the experiment and cultured with IL-2. Cytotoxicity was measured after 8 h. The slopes of linear regression for **B** were 40.4 for the non-irradiated cells and 42.5, 37.6, and 27.5 for the 2.5, 5 and 10 Gy irradiated cells, P < 0.001 for 10 Gy. The KFs for the 1:16 E:Ts are indicated in the two boxes in the middle of the figure. E:Ts are graphed on a log_10_ scale. [***p-value < 0.001 via regression analysis]

The cytotoxic capacity of NK-92 cells was measured following irradiation, either immediately or following overnight culture, to detect activity of those cells that temporarily resist the effects of lethal irradiation. We used 2.5 to 20 Gy; 10 Gy is the FDA standard for adoptive transfer. Figure 2A shows that immediately after 20 Gy irradiation, NK-92 cells retained full killing capacity. However, following overnight culture, the NK-92 effectors that were still viable showed a dose response of decreasing cytotoxic functionality as radiation dosage increased. The slopes of the linear regressions of 8 h killing (% cells killed per log_10_ increase in effector cells) that were used to calculate lytic units were substantially different between 10 Gy-irradiated cells and non-irradiated controls. For 3 experiments, the slopes were 49.5 ± 15.0 for the controls and 38.8 ± 15.8 for the irradiated cells. Paired comparisons of the slopes within each experiment were statistically significant (P < 0.01). The changes in slope are consistent with less killing per irradiated NK-92 cell. The slopes would have been similar if there were simply fewer irradiated NK-92 cells with control activity per cell.

The effects on serial killing were pronounced. At an E:T of 1:16, 35.0% of targets were killed by non-irradiated NK-92 cells (KF 5.6) compared to 9.1% killing by 10 Gy irradiated NK-92 effectors (KF 1.5), a loss of more than 2/3 of cytotoxic activity per cell (Fig. 2B, p-value < 0.001). This dose–response radiation effect on serial killing was also observed with K562 targets (not illustrated). Thus, the serial killing by the NK-92 cells was greatly decreased by FDA-approved irradiation. It should be noted that at lower doses of irradiation than the 10 Gy in current clinical practice, serial killing was still significantly impacted; 2.5 Gy irradiated NK-92 cells had a KF of 3.9 (Fig. 2B), a 30% decrease in serial cytotoxicity compared to non-irradiated NK-92 cells (p < 0.001).

Loss of cells due to radiation-induced death and loss of cytotoxic functionality act synergistically to limit the overall efficacy of irradiated NK-92 cells. When loss of serial killing is considered in combination with lower viable cell recovery, 10% of the potential NK-92 serial killing efficacy was retained 1 day after 10 Gy irradiation (Table 1). Using lytic units for comparison, as LU_50_ per 1 million NK-92 cells at 8-h, there were dose-dependent losses of overall activity following irradiation that were amplified when decreased cell survival was considered in combination with loss of function (Table 1). Activity monitored by LU_50_s 1 day after 10 Gy was consistently less than 30% of the non-irradiated NK-92 cells in replicate experiments (data not shown).

**Table 1:** Compound effects of viable cell recovery and impaired lytic activity on NK-92 cell functionality one day after irradiation*

Irradiation (Grays)	% Control viable cell recovery^+^	Serial KF	% Control KF	% Remaining functional activity based on KF^#^	LU_50_ per 10^6^ NK-92 cells	% Control lytic units	% Remaining functional activity based on lytic units^^^
0	100	5.6	100	100	815.5	100	100
2.5	53.4	3.9	69.6	37.2	419.8	51.5	27.5
5	56.8	2.5	44.6	25.4	215.9	26.5	15.0
10	39.0	1.5	26.8	10.4	23.8	2.9	1.1

*Raji 'target' cells at E:T 1:16 after 8 h. This representative experiment was comparable to two other replicate experiments

^+^Cells proliferated to increase 75–80% from time of initial culture without irradiation

^#^Calculated as the fraction representing viable cell recovery multiplied by the fraction of control KF

^^^Calculated as the fraction representing viable cell recovery multiplied by the fraction of lytic unit activity that was retained compared to untreated NK-92 cells

### Fas (CD95) receptor ligation weakens NK-92 serial killing, especially 1 day after irradiation

**Fig. 3:**
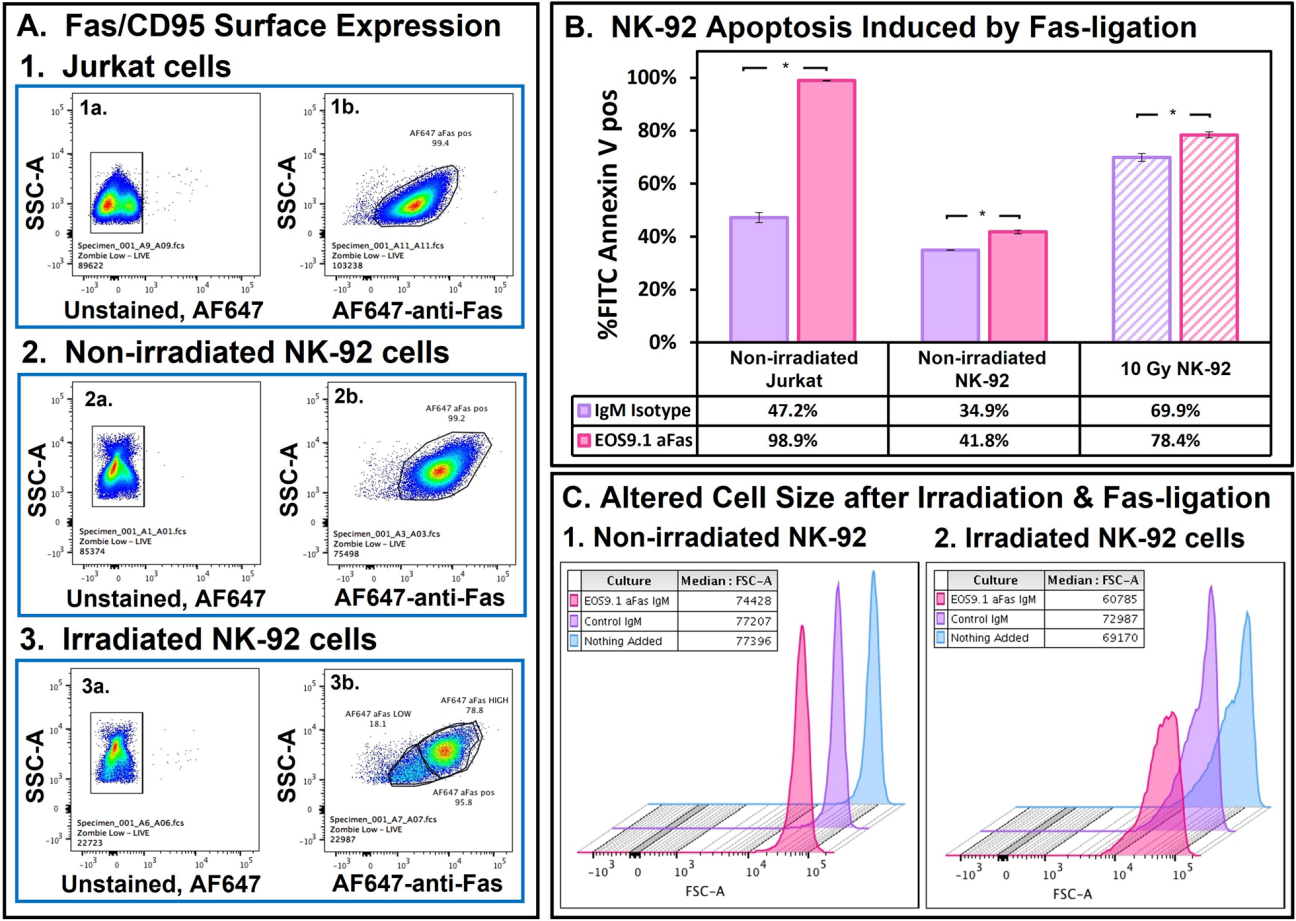
Expression of CD95 (Fas) by NK-92 cells and effects of Fas-ligation on their viability. Fas-sensitive Jurkat cells were used as positive controls for Fas expression and death after a day of Fas ligation. Dead cells are indicated by binding of FITC-labeled annexin V to phosphatidyl serine that is externalized in the plasma membranes of dying cells. **A** Surface expression of Fas/CD95.** 1** Expression of Fas by Jurkat cells. **1a** Unlabeled cells. **1b** Cells labeled with mAb anti-CD95 Fas. **2** Expression of Fas by non-irradiated NK-92 cells. 2a&2b are as indicated for **1a** & **1b**. **3** Expression of Fas by NK-92 cells irradiated with 10 Gy. 3a&3b are as indicated for **1a** &**1b**. **B** Induction of death with control IgM or IgM anti-Fas in overnight culture. By Overton subtraction, the conversion from annexin V-low to annexin V-high cells (apoptotic and necrotic) was 52% for Jurkat cells, 5.8% for non-irradiated NK-92 cells, and 9.2% for the irradiated NK-92 cells (*p < 0.05). **C** Changes in cellular size (forward scatter) in response to irradiation and Fas-ligation. **1** Non-irradiated cells cultured with media, IgM isotype, or anti-Fas IgM. **2** Cells 1 day after 10 Gy irradiation, cultured with media, IgM isotype, or anti-Fas IgM

Fas-ligand is expressed by many tumors and is a means by which these tumors can engage Fas-receptor on NK- and/or T- cells and thereby initiate “suicide” of the effector lymphocytes [32] and protection of the tumor cells. To evaluate if Fas ligation could also affect NK-92 cells, first, we monitored Fas/CD95 expression on NK-92 cells, then discovered a lack of NK-92 cell “suicide” responsiveness to Fas-ligation and, last, observed detrimental effects of Fas-ligation on serial killing. Jurkat cells that readily die after ligation of their membrane-bound Fas were used as positive controls for detection of Fas expression and induction of cellular “suicide” by anti-Fas antibodies [33]. Jurkat and NK-92 cells were stained with AF647-anti-CD95/Fas and analyzed by flow cytometry. Figure 3A shows that > 95% of Jurkat and NK-92 cells were positive for CD95/Fas. Furthermore, NK-92 cells expressed more Fas than Jurkat cells, with a higher median fluorescence index (MFI) of 6,339 that remained high after irradiation, compared to an MFI of 2,263 for Jurkat cells. It should be noted that within a day, a subpopulation of ~20% of the irradiated NK-92 cells had reduced levels of Fas (MFI 1851) (Fig. 3A, with a 43% subpopulation in a replicate experiment).

Following detection of CD95 on NK-92 cells, we then incubated non-irradiated and 10 Gy irradiated NK-92 cells with anti-Fas IgM overnight and subsequently examined these cells for apoptotic or necrotic death. Cells were stained with FITC-annexin V, which binds to phosphatidylserine externalized on apoptotic cells and also binds to the internal phosphatidyl serine of permeable necrotic cells. The Jurkat cells responded strongly to anti-Fas ligation, with 99% annexin V positive (Fig. 3B). In contrast, both non-irradiated and irradiated NK-92 cells responded weakly to Fas ligation, with a < 10% increase in annexin V-positive cells compared to the control cells (Fig. 3B). At least one third of all the annexin V-positive cells also stained positive with 7-AAD, indicating that a substantial fraction of these cells had progressed to necrosis (not illustrated). Despite NK-92 cell resistance to death following Fas ligation, anti-Fas treatment did affect the irradiated, non-necrotic NK-92 cells, in form of shrunken cells (as indicated by lower forward scatter (FSC)) (Fig. 3C).

**Fig. 4:**
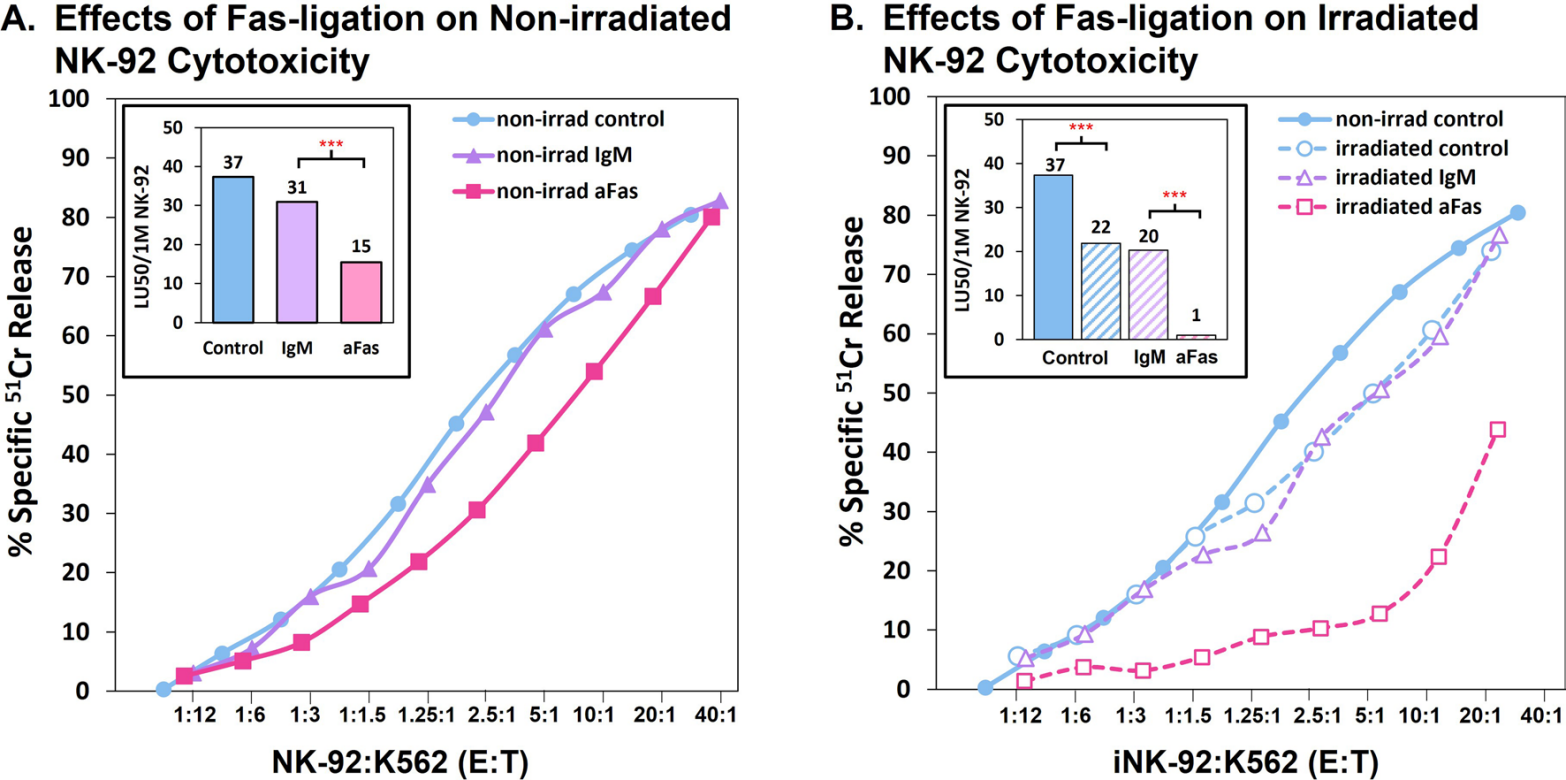
Effects of CD95 (Fas)-ligation on serial killing by non-irradiated and irradiated NK-92 cells. Non-irradiated or 10 Gy irradiated cells were cultured for 1 day with nothing, 1 ug/ml control IgM or IgM anti-Fas. Because K562 cells lack Fas, they were used as tumor ‘target’ cells to prevent addition of anti-Fas-initiated “suicide” to target cell killing by NK-92 cells. Cytotoxic activity towards K562 cells was measured after 8 h. E:T ratios are approximate. **A** Anti-Fas ligation limited to non-irradiated NK-92 cells. Insert: LU_50_/1 M non-irradiated NK-92 cell, by treatment. **B** Anti-Fas ligation of 10 Gy irradiated NK-92 cells. Insert: LU50/1 M irradiated NK-92 cell, by treatment. [***p-value < 0.001 via regression analysis]. KFs **A**, **B** The KFs calculated for a 1:1 E:T for **A**, the non-irradiated control alone, with IgM or with anti-Fas were 0.36, 0.34, and 0.22, respectively. For **B**, the KFs for the irradiated cells alone, with IgM or with anti-Fas were 0.30, 0.30 and 0.03 respectively. The loss of KF for the irradiated and anti-Fas combination exceeded both additive and synergistic effects estimated based on the losses from single treatments

In contrast to the minimal effects on NK-viability, the effects on NK-92 cytotoxic functionality were substantial after Fas-ligation. Non-irradiated or 10 Gy irradiated NK-92 cells were preincubated for 1 day without antibody, with an IgM isotype control, or with anti-Fas IgM. The cytotoxic capacity of these cells was then tested against K562 targets with the continuing presence of the antibodies in the ^51^Cr release assays. Because K562 cells lack Fas, they were used as tumor ‘target’ cells, which prevented addition of anti-Fas-initiated “suicide” to the target cell killing by NK-92 cells. Figure 4A shows that non-irradiated cells were able to maintain high cytotoxic activity following overnight Fas ligation, reaching 80% killing compared to 80–83% killing by control and IgM treated cells respectively at similar E:T ratios. By LU_50_, anti-Fas treated NK-92 cells retained ~ 50% activity compared to the IgM isotype control (insert, Fig. 4A). Following 10 Gy irradiation, however, anti-Fas treated NK-92 cytotoxic activity was significantly decreased, reaching 44% killing compared to 74–77% killing by control and IgM treated cells respectively (Fig. 4B). The insert of Fig. 4B illustrates that anti-Fas treated, 10 Gy irradiated NK-92 cells retained just < 5% activity by LU_50_ compared to control cells.

### Irradiated NK-92 cells are susceptible to killing by primary blood NK cells (bNK) and IL-2 lymphokine-activated killer (LAK) cells

While one would like to know if primary host NK cell attack of adoptively transferred NK-92 tumor cells has effects on the cytotoxic functionality of NK-92 cells (even if the NK-92 cells were to resist attack), the investigation is technically stymied because both the primary NK and NK-92 cells would be concurrently mediating ‘target cell’ death. It would be impossible to sort out the contributions of each cell. Instead, we simply determined if irradiated NK-92 cells could be attacked by the patients’ own NK cells, which would be another means by which efficacy of NK-92 cell adoptive therapy could be compromised in vivo. The susceptibility of non-irradiated NK-92 cells to primary NK and IL-2 induced LAK cell attack has been well-documented [34, 35]. We wanted to assess if this susceptibility was increased for irradiated NK-92 cells. This possibility is supported by the induction of stress ligands after irradiation and these ligands can serve as recognition molecules for NK cells [36].

In preliminary experiments, we tested the ability of resting bNK or LAK cells to kill NK-92 cells. The data are presented as PBMC:NK-92, effector: target (E:T) ratios. The E:T ratios differ among the experiments because the cell yields varied with the donors. We started with the highest possible E:Ts. We also addressed whether IL-2 induced NK cells to divide significantly and result in more LAK cells than bNK cells within the PBMCs. We used Trucounts® with flow cytometry to determine bNK and LAK frequencies (see Methods) and found that the frequencies were similar after short term culture. We used K562 cells as control ‘targets’ to confirm that the healthy donors’ bNK and LAK cells had good cytotoxic activity, whether or not their NK cells killed NK-92 cells. The NK-92 target cells were either non-irradiated or irradiated with 10 Gy and cultured overnight before assay.

**Fig. 5:**
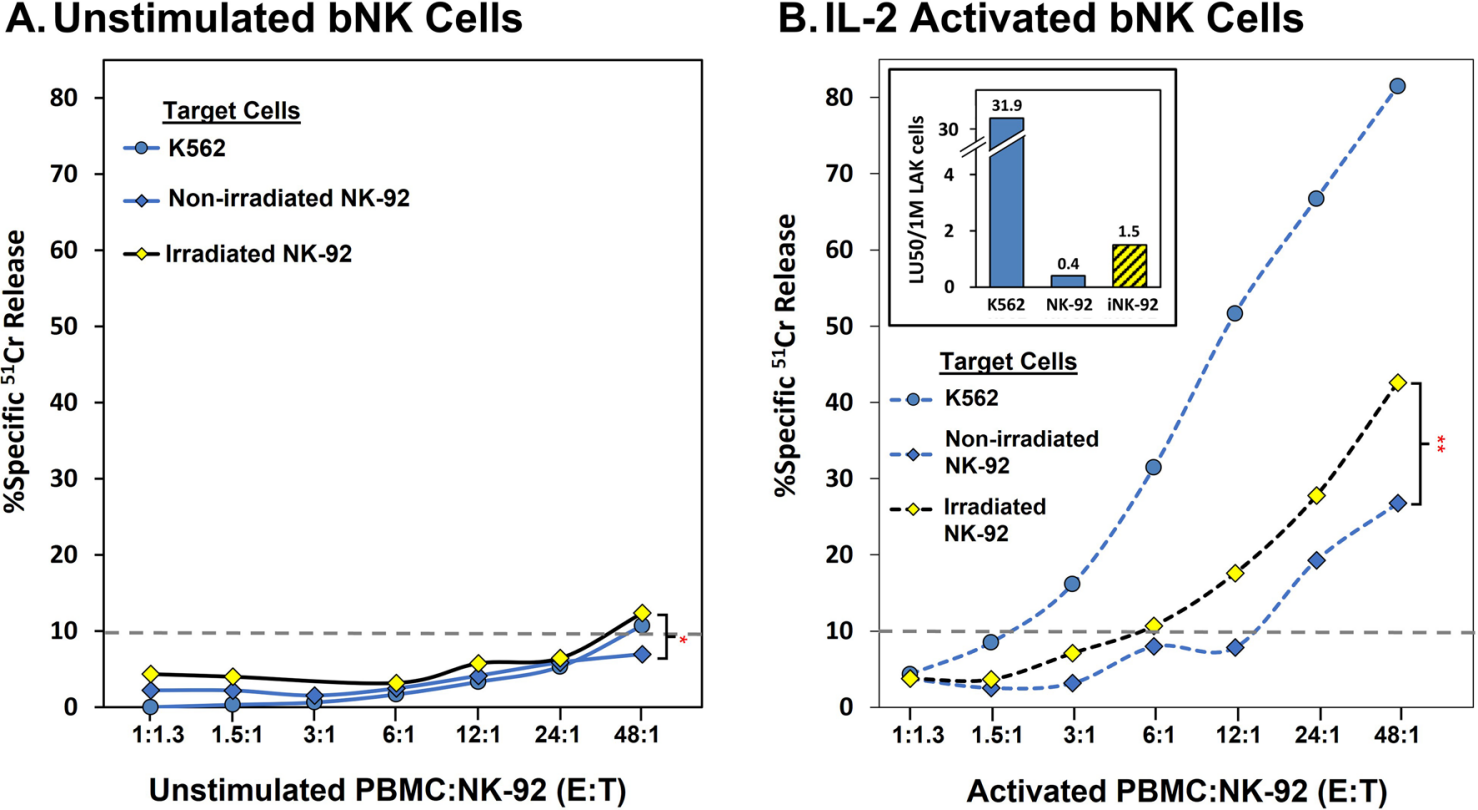
Killing of NK-92 cells by either resting or activated primary blood NK cells. The susceptibility of NK-92 tumor cells to attack by primary peripheral blood NK cells from healthy donors was investigated using NK-92 cells that were either non-irradiated or were irradiated with 10 Gy and then cultured for 1 day. The primary NK cells were either resting or activated by 3 days culture with 1000 u/ml IL-2. Cytotoxicity was measured using 4-h ^51^Cr-release assays; the gray dashed line reflects the 10% threshold for positive killing. The results illustrated represent concurrent assays made with E:Ts of viable effector NK cells from a single donor SC-0975 and are representative of 4 experiments each with a different donor. **A** Killing of NK-92 cells by resting primary NK cells. LU_50_s were excluded for unstimulated NK cells because killing was over 10% for only the highest E:T. **B** Killing of NK-92 cells by activated primary NK cells. For both bNKs and LAKs, the cytotoxicity towards non-irradiated versus irradiated NK-92 cells was significantly different at the highest E:T (student t-test, *p < 0.03, **p < 0.005). Insert for **B** LU50/1 M LAK bNK calculated using TruCOUNT™ beads (Gating—Additional file 2: Fig. S1)

Figure 5 illustrates that irradiated NK-92 cells are especially vulnerable to attack by LAK cells. After culture, bNK cells of this donor had low activity, regardless of the targets. In contrast, the LAK cells were very active, preferentially killing irradiated NK-92 cells compared to non-irradiated NK-92 cells. In concordance with the lack of bNK activity of the donor of Fig. 5, unstimulated bNK cells from other donors also had low cytotoxic activities, as indicated in Table 2. LAK cells of donor SC-6617 were able to kill both non-irradiated and irradiated NK-92 cells, also with a clear increase in LAK *vs.* bNK anti-NK-92 cell killing (Table 2). These limited experiments indicate that after irradiation, NK-92 cells retain and/or increase ligands for NK attack and thus are susceptible to innate immune elimination in vivo, indicating a third consideration that could be addressed by avoiding irradiation and/or by genetic engineering.

**Table 2:** Primary blood NK and LAK killing of NK-92 cells at 4 h

NK activation state	Donor #	E:T (PBMC:NK-92)	^51^Cr 'Target' cells^+^		
			Non-irradiated NK-92	10 Gy irradiated NK-92	K562
Unstimulated	SC-0394	107:1	7%*	15%	76%
	SC-3663	33.5:1	2%*	6%*	23%
	SC-6617	60:1	8%*	19%	13%
	SC-0975	48:1	7%*	12%	11%
IL-2 LAK	SC-0394	23:01	8%*	ND	96%
	SC-3663	15.2:1	1%*	4%*	47%
	SC-6617	55:1	17%	31%	78%
	SC-0975	42:1	27%	43%	82%

^**+**^%Specific Release. Data are available in Additional file 4: Fig. S2 for the E:T titrations

^*^Indicates cytotoxicity below the positive killing threshold of 10%

## Discussion

In this study we demonstrated the remarkable serial killing potential of NK-92 cells towards several ‘target’ cell types and the potential for gamma-irradiation to affect this killing. We also tested NK-92 cell functionality remaining after Fas-ligation and documented NK-92 susceptibility to attack by circulating bNK cells, which are two potential limitations of adoptive cell therapy. These findings suggest that NK-92 cells have immense potential in adoptive cancer immunotherapy but should be carefully optimized before infusion into patients to ensure greatest therapeutic efficacy.

Serial killing by NK-92 cells has been documented before; however, we directed our attention to potential in vivo inhibitory effects on serial killing that are absent from standard in vitro NK assays (e.g., FasL on myeloid cells). A previous study focused on the development of a droplet-based cytotoxicity assay that utilized a lowest E:T of 1:3 and showed that ~ 50% of observed NK-92 cells are able to serially kill two or more K562 targets in 12 h [37]. Serial cytotoxicity has also been observed with time-lapse cinematography using *genetically modified*, IL-2 producing NK92-MI cells. This study indicated that one NK-92 cell could kill as many as 14 target HeLa cells over 6 h [30]. Many studies have used standard radioactive release assays to characterize NK-92 killing towards various cell types, but, without an excess of ‘targets’, these assays were unable to address NK-92 serial cytotoxicity. Our results confirm that NK-92 cells are serial killers and show that serial killing is target cell type dependent, with Raji and Daudi targets reaching KFs > 10, while K562 targets are far less susceptible to serial killing with KFs < 3.0 (Fig. 1).

This variation in killing for different ‘target’ cells, as determined by KFs, may be due to differences in ligands on target cells that engage the diverse activation receptors of NK-92 cells. One possible explanation for low killing towards K562 cells is that NK-92 cells poorly express the receptor NKG2D, in contrast to high NKG2D-expressing KHYG-1 cell line that kills K562 cells much more effectively [38]. Furthermore, K562s produce the granzyme B inhibitor PI-9, making them less susceptible to killing via granzyme B, which is predominately used by NK-92 cells but overcome by granzyme M used by KHGY-1 [39]. It is possible that cleavage of NKG2D ligands by metalloproteinases such as ADAM10 [40] combined with NKG2D downregulation during cytotoxic activation [41] affects NK-92 killing of K562s. This loss of NKG2D may also explain the plateau in killing that we observed towards K562 after 6 h, and which has been observed for up to 12 h elsewhere [37]. A limitation of the KF method [5] to detect serial killing is that it measures the simple average number of targets killed per effector, rather than identifying the fraction of effectors engaged in killing within the NK cell population. Regardless, the KF method is still optimal (in terms of statistical validity, time, labor, and cost savings) to screen treatments used prior to adoptive cell transfer for their effects on serial killing.

In this report, we shed new light on potential limitations to NK-92 cell-mediated serial killing and therapeutic efficacy, specifically following irradiation, a current clinical practice preceding adoptive transfer. Prior studies have investigated the effects of irradiation on NK-92 cell-mediated killing; however, these studies used shorter cytotoxicity assays with different target cells and reported that NK-92 cytotoxic capacity is mostly retained for at least 24 h following irradiation [22, 42]. Another study, using haNK cells 24-h post irradiation saw an increase in cytotoxicity toward multiple carcinoma cell lines compared to haNK cell killing immediately post irradiation [43]. These findings are in contrast to our findings with unmodified NK-92 cells, which show decreases in killing toward Raji cells 1 day after irradiation (Fig. 2) as well as K562 cells (*unpublished*). Possible explanations for the differences between these findings include haNK cell endogenous expression of IL-2, while our cells were supplemented with 1000 U/ml IL-2, lymphoid versus carcinoma target cells, as well as the 18-h release assays used for the haNK cells, which may allow for detection of longer-term killing potential than our 8-h release assays. It should be recognized that for definitive assessment of serial killing and its losses, two other approaches, time-lapse cinematography and microchip assays with single effector cells with multiple target cells in individual wells [8, 30, 44, 45], have advantages over KF frequencies. These alternative approaches can discriminate between slower killing by all effector cells vs. killing by a combination of both totally inactive and fully active cells.

In this report, we have also extended the effects of irradiation to effects on cytotoxicity after Fas-ligation and to NK-92 cellular susceptibility to potential attack by patient NK cells. First, we observed a decrease in NK-92 cell viability and viable cell recovery (Table 1), as well as a consistent decrease in serial cytotoxicity 1 day after irradiation, even at lower doses of 2.5–5 Gy (Fig. 2). Notably, this decrease in killing was absent when NK-92 cells were assayed immediately following up to 20 Gy irradiation. This initial retention of activity indicates that therapeutic cell lines should be used immediately post irradiation to maximize cytotoxicity in vivo*,* as in the design of one phase I trial [21]. Ideally, an alternative anti-proliferative approach for cell lines used in adoptive therapies would be used.

The profound effects of irradiation on NK-92 cytotoxic capacity indicate that radiation effects extend beyond DNA damage and likely include direct damage to proteins [46, 47]. Ionizing radiation produces radiolysis products, such as reactive oxygen species that inactivate proteome functions including those involved in killing and DNA repair [42]. Low energy electron irradiation, as an alternative to gamma irradiation, inhibits NK-92 cell proliferation while maintaining higher cytotoxic capacity and for longer periods of time and could therefore be considered for clinical applications [42]. This report also indicates that 2-h after 10 Gy gamma irradiation, there is lower expression of genes encoding multiple pathways that are critical to cell-mediated cytotoxicity [42]. Considering the additional impact of direct proteome damage by irradiation, alternative treatments such as induction of genetically introduced type II restriction enzymes and pretreatment of cells with certain topoisomerase inhibitors (Hudig et al., unpublished results) that only inflict damage to DNA could be used prior to adoptive transfer [47].

We discovered an Achille’s heel for irradiated NK-92 cells, Fas/CD95, which has previously been noted on the majority of activated NK cells [48, 49] and on NK-92 cells [50]. Despite high expression of CD95, anti-Fas antibodies alone failed to affect proliferation or to initiate death of non-irradiated NK-92 cells within 1 day, even though the non-irradiated cells did respond to Fas-ligation by shrinking in size. One possible explanation for the NK-92 cell’s low sensitivity to death after anti-Fas ligation is that there are two pathways of Fas-mediated death, one of which relies on mitochondrial signal amplification. This type II, intrinsic pathway is slow and readily inhibited by expression of the Bcl-2 family of apoptotic proteins [32, 51]. Another possible explanation is that NK-92 cells may express wild-type PI-9, which inhibits the caspase-dependent Fas/FasL-mediated death pathways [52]. Intrinsic resistance to Fas-ligation is also indicated by evidence that NK-92 cells constitutively produce soluble Fas ligand [42].

Even though the NK-92 cells resisted death by Fas, they did respond with decreased cytotoxic activity. Fas-ligation alone could decrease NK-92 cytotoxicity to Raji cells, but these effects were always two-fold or less for non-irradiated effector cells. However, for irradiated NK-92 cells the anti-Fas effect was remarkably stronger, with just 10% or less of control killing remaining. In synergy, Fas-ligation and irradiation profoundly reduced cytotoxicity (Fig. 4). One possible explanation for this synergistic effect, seen with cytotoxicity but not with viability, is that the cell shrinkage that occurred with Fas-ligated irradiated, non-necrotic NK-92 cells impaired their activity. This shrinkage, that was absent from Fas-ligated non-irradiated cells, is related to dehydration and has been reported as an early indicator of cell death [53].

These findings suggest a serious risk for engagement of CD95 as a mechanism to hamper NK-92 cell therapeutic efficacy in vivo, especially if a patient’s tumor cells express the counter Fas-ligand (Fas-L/CD178). After irradiation, NK-92 cells appear to have normal viability in the face of Fas-mediated death receptor ligation but are considerably less-effective killers. A logical next step could be to remove CD95 from NK-92 cells in order to reduce their susceptibility to rapid death via the Fas pathway. Recent advances in CRISPR/Cas-9 have made the methodology a more efficient way to genetically engineer NK-92 cells, including the implementation of multiple genetic changes at one time [54].

Having discovered that irradiation affects NK-92 cell susceptibility to Fas-ligation, we queried if irradiation would also make NK-92 cells more vulnerable to attack when encountered by patient NK cells. We found that irradiated NK-92 cells are susceptible to attack by both unstimulated and IL-2 LAK bNK, whereas non-irradiated NK-92 cells were more resistant to killing (Table 2). These results contrast with previous reports in which substantial killing to non-irradiated NK-92 cells (comparable to K562) was observed [34, 35]. A technical consideration may contribute to these differences: the IL-2 concentration used to maintain the susceptible NK-92 cells was 20 U/ml, while we used 1000 U/ml IL-2. Our results are preliminary due to a limited number of NK cell donors but do indicate that, after irradiation, NK-92 cells may become more sensitive to attack by circulating NK cells. This NK -mediated attack could potentially be further promoted by antibody-dependent cell-mediated cytotoxicity (ADCC) supported by IgG antibodies that patients develop to NK-92 cell MHC class I proteins [21]. We suggest that increased sensitivity to host cell attack be monitored whenever NK-92 cells are genetically modified or are treated before adoptive transfer.

Our research was limited to the cytotoxic NK line NK-92, which is only one of several lines that are available for immunotherapies (Additional file 1: Table S1). To the best of our knowledge, these other immortalized cell lines and induced pluripotent NK cells have yet to be characterized for serial killing and for the effects of irradiation combined with Fas ligation. Our study is also limited in that all assays were conducted in vitro. Nonetheless, we were able to underscore the importance of serial killing as a critical variable that may be compromised by pretreatments such as irradiation and by in vivo conditions such as intratumor Fas ligand and bNK attack. The research indicates that other cells lines should be similarly evaluated for potential effects on serial killing. Tumor counter-ligands other than Fas that stimulate NK inhibitory receptors may also profoundly compromise serial killing, a possibility that is yet to be explored. A broad implication is that it may become clinically worthwhile to genetically profile ligands of a tumor environment that affect NK serial killing to select the best NK cell line for immunotherapy.

## Conclusions

In conclusion, we indicate here that non-irradiated NK-92 cells are more effective than irradiated NK-92 cells in three ways: increased serial killing, resistance to Fas-ligation, and resistance to attack by NK or LAK cells. Our findings suggest that studies with non-irradiated NK-92 cells in murine models may have overlooked these three limitations and that lesser outcomes would have occurred with irradiated NK-92 cells. Future studies in mice should include NK-92 pretreatments that parallel the pretreatments used for clinical trials. Our data warrant urgent changes for clinical immunotherapy. Of greatest importance is replacement of irradiation with alternative methods to prevent NK-92 tumor engraftment and, potentially, deletion of Fas from the NK-92 cells.

## Supplementary Information

**Additional file 1: Table S1.** NK cell lines derived from malignancies or from blood NK cells of healthy donors. List of NK derived cell lines, including origin, year published, PMID, and expression of CD16.

**Additional file 2**: **Figure S1.** Flow cytometry to determine the number of primary NK cells within peripheral blood mononuclear cells. A. Gating sequence. 1. First, beads were gated by high Pacific Orange signal. 2. Cells without debris were gated by forward scatter (FSC-A) v side scatter (SSC-A). 3. Single cells (without doublets) were gated by SSC height versus SSC width. 4. All cells were gated on by Pacific Blue anti-CD45. 5. Live cells were gated by Boolean not gate, taking 7-AAD positive cells as dead cells. 6. T-cells were gated out by Boolean not gate of FITC anti-CD3 positive T-cells. 7. NK cells were gated on as AF647 anti-CD244 positive cells. CD244 is expressed by CD8+ T-cells (gated out with CD3) and all NK cells [24]. 8. NK cell staining for BV711 anti-CD16/CD56. B. Expression of CD16A & CD56 by resting and IL-2 activated (LAK) bNK cells. Light blue samples are the unstained control cells respective for each group. Red samples are stained CD16A and/or CD56. 1. Unstimulated bNK cells. 2. Interleukin-2 activated LAK bNK cells.

**Additional file 3: Table S2.** NK -92 KFs at E:T 1:16 after 8 h. Documentation of experimental variability of NK-92 serial killing toward Raji, Daudi and K562 ‘target’ cells.

**Additional file 4**: **Figure S2**. Killing of NK-92 cells by either resting or activated primary blood NK cells. The susceptibility of NK-92 cells to attack by primary peripheral blood NK cells from healthy donors was investigated using NK-92 cells that were either non-irradiated or were irradiated with 10 Gy and then cultured for 1 day. The primary NK cells were either resting or activated by 3 days culture with 1000 u/ml IL-2. Cytotoxicity was measured using 4-h ^51^Cr-release assays; the horizontal gray dashed line reflects the 10% threshold for positive killing. The results illustrated represent assays of multiple donors across multiple experiments.

## Data Availability

The data that support the findings of this study are included in this published article and its supplementary information files. Additional data are available from the corresponding author on reasonable request.

## Abbreviations

ADCC: Antibody dependent cellular cytotoxicity
CAR: Chimeric antigen receptor
E:T: Effector to target ratio
IL-2: Interleukin-2
KF: Killing frequency
LAK: Lymphokine-activated killer
LU_50_: Lytic units as the number of cells required to kill 50% of the ‘target’ cells
mAb: Monoclonal antibody
NK: Natural killer, including bNK as primary blood NK cell
PBMC: Peripheral blood mononuclear cells

## References

[1] Miller JS, Lanier LL (2019). Natural killer cells in cancer immunotherapy. Ann Rev Cancer Biol..

[2] Hodgins JJ, Khan ST, Park MM, Auer RC, Ardolino M (2019). Killers 2.0: NK cell therapies at the forefront of cancer control. J Clin Investig.

[3] Heipertz EL, Zynda ER, Stav-Noraas TE, Hungler AD, Boucher SE, Kaur N (2021). Current perspectives on “Off-The-Shelf” allogeneic NK and CAR-NK cell therapies. Front Immunol.

[4] Isaaz S, Baetz K, Olsen K, Podack E, Griffiths GM (1995). Serial killing by cytotoxic T lymphocytes: T cell receptor triggers degranulation, re-filling of the lytic granules and secretion of lytic proteins via a non-granule pathway. Eur J Immunol.

[5] Bhat R, Watzl C (2007). Serial killing of tumor cells by human natural killer cells—enhancement by therapeutic antibodies. PLoS ONE.

[6] Vanherberghen B, Olofsson PE, Forslund E, Sternberg-Simon M, Khorshidi MA, Pacouret S (2013). Classification of human natural killer cells based on migration behavior and cytotoxic response. Blood.

[7] Perussia B, Trinchieri G (1981). Inactivation of natural killer cell cytotoxic activity after interaction with target cells. J Immunobiol.

[8] Romain G, Senyukov V, Rey-Villamizar N, Merouane A, Kelton W, Liadi I (2014). Antibody Fc engineering improves frequency and promotes kinetic boosting of serial killing mediated by NK cells. Blood.

[9] Srpan K, Ambrose A, Karampatzakis A, Saeed M, Cartwright ANR, Guldevall K (2018). Shedding of CD16 disassembles the NK cell immune synapse and boosts serial engagement of target cells. J Cell Biol.

[10] Klingemann H, Boissel L, Toneguzzo F (2016). Natural killer cells for immunotherapy—advantages of the NK-92 cell line over blood NK cells. Front Immunol.

[11] Vivier E, Raulet DH, Moretta A, Caligiuri MA, Zitvogel L, Lanier LL (2011). Innate or adaptive immunity? The example of natural killer cells. Science.

[12] Gunesch JT, Angelo LS, Mahapatra S, Deering RP, Kowalko JE, Sleiman P (2019). Genome-wide analyses and functional profiling of human NK cell lines. Mol Immunol.

[13] Yang HG, Kang MC, Kim TY, Hwang I, Jin HT, Sung YC (2019). Discovery of a novel natural killer cell line with distinct immunostimulatory and proliferative potential as an alternative platform for cancer immunotherapy. J Immunother Cancer.

[14] Solocinski K, Padget MR, Fabian KP, Wolfson B, Cecchi F, Hembrough T (2020). Overcoming hypoxia-induced functional suppression of NK cells. J Immunother Cancer.

[15] Suck G, Odendahl M, Nowakowska P, Seidl C, Wels WS, Klingemann HG (2016). NK-92: an ‘off-the-shelf therapeutic’ for adoptive natural killer cell-based cancer immunotherapy. Cancer Immunol Immunother.

[16] Jochems C, Hodge JW, Fantini M, Tsang KY, Vandeveer AJ, Gulley JL (2017). ADCC employing an NK cell line (haNK) expressing the high affinity CD16 allele with avelumab, an anti-PD-L1 antibody. Int J Cancer.

[17] Fabian KP, Padget MR, Donahue RN, Solocinski K, Robbins Y, Allen CT (2020). PD-L1 targeting high-affinity NK (t-haNK) cells induce direct antitumor effects and target suppressive MDSC populations. J Immunother Cancer.

[18] Arai S, Meagher R, Swearingen M, Myint H, Rich E, Martinson J (2008). Infusion of the allogeneic cell line NK-92 in patients with advanced renal cell cancer or melanoma: a phase I trial. Cytotherapy.

[19] Tonn T, Schwabe D, Klingemann HG, Becker S, Esser R, Koehl U (2013). Treatment of patients with advanced cancer with the natural killer cell line NK-92. Cytotherapy.

[20] Burger M. Intracranial injection of NK-92/5.28.z cells in patients with recurrent HER2-positive glioblastoma (CAR2BRAIN). Identifier NCT03383978. National Library of Medicine U.S.; 2017.

[21] Williams BA, Law AD, Routy B, denHollander N, Gupta V, Wang XH (2017). A phase I trial of NK-92 cells for refractory hematological malignancies relapsing after autologous hematopoietic cell transplantation shows safety and evidence of efficacy. Oncotarget.

[22] Klingemann H-G, Wong E, Maki G (1996). A cytotoxic NK-cell line (NK-92) for ex vivo purging of leukemia from blood. Biol Blood Marrow Transpl.

[23] Golstein P. Cytotoxicity, mechanisms of encyclopedia of immunology. 1998;732–4.

[24] McNerney ME, Lee KM, Kumar V (2005). 2B4 (CD244) is a non-MHC binding receptor with multiple functions on natural killer cells and CD8+ T cells. Mol Immunol.

[25] Pross HF, Maroun JA (1984). The standardization of NK cell assays for use in studies of biological response modifiers. J Immunol Methods.

[26] Bryant J, Day R, Whiteside TL, Herberman RB (1992). Calculation of lytic units for the expression of cell-mediated cytotoxicity. J Immunol Methods.

[27] Overton WR (1988). Modified histogram subtraction technique for analysis of flow cytometry data. Cytometry.

[28] Yan Y, Steinherz P, Klingemann HG, Dennig D, Childs BH, McGuirk J (1998). Antileukemia activity of a natural killer cell line against human leukemias. Clin Cancer Res.

[29] Alter G, Malenfant JM, Altfeld M (2004). CD107a as a functional marker for the identification of natural killer cell activity. J Immunol Methods.

[30] Choi PJ, Mitchison TJ (2013). Imaging burst kinetics and spatial coordination during serial killing by single natural killer cells. Proc Natl Acad Sci USA.

[31] Xu Y, Zhou S, Lam YW, Pang SW (2017). Dynamics of natural Killer cells cytotoxicity in Microwell arrays with connecting channels. Front Immunol.

[32] Timmer T, de Vries EGE, de Jong S (2002). Fas receptor-mediated apoptosis: a clinical application?. J Pathol.

[33] Matsui H, Tsuji S, Nishimura H, Nagasawa S (1994). Activation of the alternative pathway of complement by apoptotic Jurkat cells. FEBS Lett.

[34] Bergman H, Sissala N, Hägerstrand H, Lindqvist C (2020). Human NK-92 cells function as target cells for human NK Cells—implications for CAR NK-92 therapies. Anticancer Res.

[35] Bergman H, Lindqvist C (2021). Human IL-15 inhibits NK cells specific for human NK-92 cells. Anticancer Res.

[36] Ames E, Canter RJ, Grossenbacher SK, Mac S, Smith RC, Monjazeb AM (2015). Enhanced targeting of stem-like solid tumor cells with radiation and natural killer cells. OncoImmunology..

[37] Antona S, Platzman I, Spatz JP (2020). Droplet-based cytotoxicity assay: implementation of time-efficient screening of antitumor activity of natural killer cells. ACS Omega.

[38] Suck G, Branch DR, Smyth MJ, Miller RG, Vergidis J, Fahim S (2005). KHYG-1, a model for the study of enhanced natural killer cell cytotoxicity. Exp Hematol.

[39] Classen CF, Ushmorov A, Bird P, Debatin KM (2004). The granzyme B inhibitor PI-9 is differentially expressed in all main subtypes of pediatric acute lymphoblastic leukemias. Haematologica.

[40] Zingoni A, Vulpis E, Loconte L, Santoni A (2020). NKG2D ligand shedding in response to stress: role of ADAM10. Front Immunol.

[41] Molfetta R, Quatrini L, Santoni A, Paolini R (2017). Regulation of NKG2D-dependent NK Cell functions: the Yin and the Yang of receptor endocytosis. Int J Mol Sci.

[42] Walcher L, Kistenmacher AK, Sommer C, Böhlen S, Ziemann C, Dehmel S (2021). Low energy electron irradiation is a potent alternative to gamma irradiation for the inactivation of (CAR-)NK-92 cells in ATMP manufacturing. Front Immunol.

[43] Jochems C, Hodge JW, Fantini M, Fujii R, Maurice Morillon YI, Greiner JW (2016). An NK cell line (haNK) expressing high levels of granzyme and engineered to express the high affinity CD16 allele. Oncotarget.

[44] Guldevall K, Brandt L, Forslund E, Olofsson K, Frisk TW, Olofsson PE (2016). Microchip screening platform for single cell assessment of NK cell cytotoxicity. Front Immunol.

[45] Kim SE, Kim H, Doh J (2019). Single cell arrays of hematological cancer cells for assessment of lymphocyte cytotoxicity dynamics, serial killing, and extracellular molecules. Lab Chip.

[46] Krisko A, Radman M (2010). Protein damage and death by radiation in *Escherichia coli* and *Deinococcus radiodurans*. Proc Nat Acad Sci USA..

[47] Radman M (2016). Protein damage, radiation sensitivity and aging. DNA Repair.

[48] Robertson MJ, Manley TJ, Pichert G, Cameron C, Cochran KJ, Levine H (1995). Functional consequences of APO-1/fas (CD95) antigen expression by normal and neoplastic hematopoietic cells. Leuk Lymphoma.

[49] Medvedev AE, Johnsen AC, Haux J, Steinkjer B, Egeberg K, Lynch DH (1997). Regulation of Fas and Fas-ligand expression in NK cells by cytokines and the involvement of FAS-ligand in NK/LAK cell-mediated cytotoxicity. Cytokine.

[50] Han R, Wu WQ, Wu XP, Liu CY (2015). Effect of total flavonoids from the seeds of Astragali complanati on natural killer cell function. J Ethnopharmacol.

[51] Barnhart BC, Alappat EC, Peter ME (2003). The CD95 Type I/Type II model. Semin Immunol.

[52] Cunningham TD, Jiang X, Shapiro DJ (2007). Expression of high levels of human proteinase inhibitor 9 blocks both perforin/granzyme and Fas/Fas ligand-mediated cytotoxicity. Cell Immunol.

[53] Wlodkowic D, Skommer J, Darzynkiewicz Z (2012). Cytometry of apoptosis. Historical perspective and new advances. Exp Oncol.

[54] Huang RS, Shih HA, Lai MC, Chang YJ, Lin S (2020). Enhanced NK-92 cytotoxicity by CRISPR genome engineering using Cas9 ribonucleoproteins. Front Immunol.

