# Supplementary material for: Optimizing NK-92 serial killers: gamma irradiation, CD95/Fas-ligation, and NK or LAK attack limit cytotoxic efficacy
Source: J Transl Med. 2022 Apr 2;20:151. doi: 10.1186/s12967-022-03350-6 (PMC8976335; doi:10.1186/s12967-022-03350-6)
Supplement: Supplementary file 1 — Additional file 1: Table S1. NK cell lines derived from malignancies or from blood NK cells of healthy donors. List of NK derived cell lines, including origin, year published, PMID, and expression of CD16. [file 12967_2022_3350_MOESM1_ESM.pdf]

Table S1. NK cell lines derived from malignancies or from blood NK cells of healthy donors

Cell Lines	Origin	Published	PMID	CD16
NK3.3*	Peripheral blood	1982	6982944	+
YT	Lymphoblastic lymphoma	1985	2578514	+
YT2C2 & YTC3	Subclone of YT	1992	1313126	+
YTS	Subclone of YT	2006	16801532	-
NK-92	LGL - lymphoma	1994	8152260	-
NKL	LGL - leukemia	1996	8599969	+
NK-YS	NK - nasal lymphoma	1998	9694726	-
KHYG-1	LGL - leukemia	2000	10803526	-
SNK-6	NK - nasal lymphoma	2001	11157488	-
IMC-1	LGL - leukemia	2004	14687623	+
NKG	LGL - lymphoma	2011	21669033	-
MJ23 plus 4*	Cord or peripheral blood	2014	23787393	+
NK101	NK/T cell lymphoma	2019	31126350	-

*NK3.3 and other indicated NK-derived cell lines were derived from healthy donor cells
