# Supplementary material for: Optimizing NK-92 serial killers: gamma irradiation, CD95/Fas-ligation, and NK or LAK attack limit cytotoxic efficacy
Source: J Transl Med. 2022 Apr 2;20:151. doi: 10.1186/s12967-022-03350-6 (PMC8976335; doi:10.1186/s12967-022-03350-6)
Supplement: Supplementary file 2 — Additional file 2: Figure S1. Flow cytometry to determine the number of primary NK cells within peripheral blood mononuclear cells. A. Gating sequence. 1. First, beads were gated by high Pacific Orange signal. 2. Cells without debris were gated by forward scatter (FSC-A) v side scatter (SSC-A). 3. Single cells (without doublets) were gated by SSC height versus SSC width. 4. All cells were gated on by Pacific Blue anti-CD45. 5. Live cells were gated by Boolean not gate, taking 7-AAD positive cells as dead cells. 6. T-cells were gated out by Boolean not gate of FITC anti-CD3 positive T-cells. 7. NK cells were gated on as AF647 anti-CD244 positive cells. CD244 is expressed by CD8+ T-cells (gated out with CD3) and all NK cells [24]. 8. NK cell staining for BV711 anti-CD16/CD56. B. Expression of CD16A & CD56 by resting and IL-2 activated (LAK) bNK cells. Light blue samples are the unstained control cells respective for each group. Red samples are stained CD16A and/or CD56. 1. Unstimulated bNK cells. 2. Interleukin-2 activated LAK bNK cells. [file 12967_2022_3350_MOESM2_ESM.pdf]

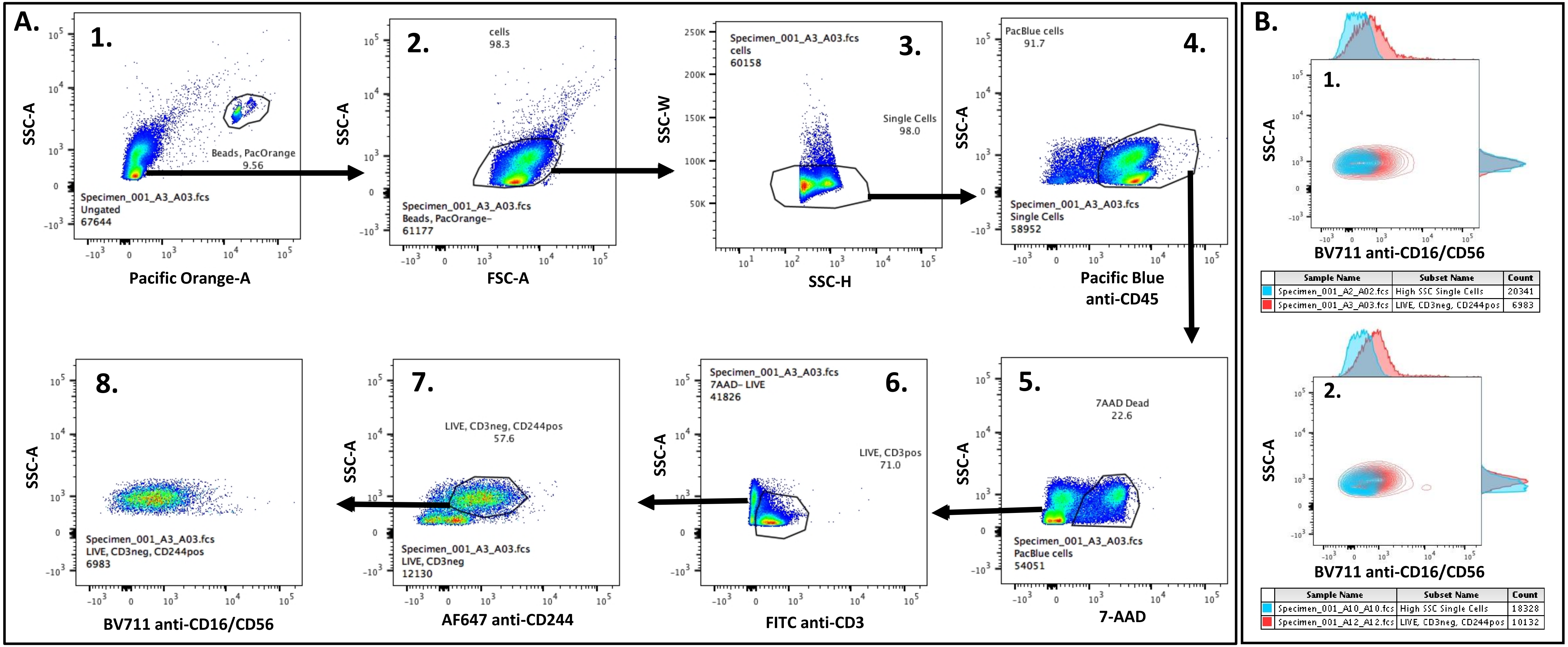

Figure S1. Flow cytometry to determine the number of primary NK cells within peripheral blood mononuclear cells. A. Gating sequence. 1. First, beads were gated by high Pacific Orange signal. **2.** Cells without debris were gated by forward scatter (FSC-A) v side scatter (SSC-A). **3.** Single cells (without doublets) were gated by SSC height versus SSC width. **4.** All cells were gated on by Pacific Blue anti-CD45. **5.** Live cells were gated by Boolean not gate, taking 7-AAD positive cells as dead cells. **6.** T-cells were gated out by Boolean not gate of FITC anti-CD3 positive T-cells. **7.** NK cells were gated on as AF647 anti-CD244 positive cells. CD244 is expressed by CD8+ T-cells (gated out with CD3) and all NK cells [see ref 22]. **8.** NK cell staining for BV711 anti-CD16/CD56. **B. Expression of CD16A & CD56 by resting and IL-2 activated (LAK) bNK cells.** Light blue samples are the unstained control cells respective for each group. Red samples are stained CD16A and/or CD56. **1.** Unstimulated bNK cells. **2.** Interleukin-2 activated LAK bNK cells.
