# Supplementary material for: Optimizing NK-92 serial killers: gamma irradiation, CD95/Fas-ligation, and NK or LAK attack limit cytotoxic efficacy
Source: J Transl Med. 2022 Apr 2;20:151. doi: 10.1186/s12967-022-03350-6 (PMC8976335; doi:10.1186/s12967-022-03350-6)
Supplement: Supplementary file 3 — Additional file 3: Table S2. NK -92 KFs at E:T 1:16 after 8 h. Documentation of experimental variability of NK-92 serial killing toward Raji, Daudi and K562 ‘target’ cells. [file 12967_2022_3350_MOESM3_ESM.pdf]

Table S2. NK-92 KFs at E:T 1:16 after 8 Hours					
**Target**	**Average**	**Min**	**Max**	**N**	**Stdev**
Raji	6.7	3.0	9.5	11	2.2
Daudi	6.8	6.5	8.5	3	1.6
K562	2.0	0.3	3.5	4	1.4

Data are based on calculations from both observed results and extrapolations based on the linear equation from LU_{50} graphs. For Rajis & Daudis, 1 experiment is extrapolated data. For K562s, 2 experiments are extrapolated data.
