# Supplementary material for: Optimizing NK-92 serial killers: gamma irradiation, CD95/Fas-ligation, and NK or LAK attack limit cytotoxic efficacy
Source: J Transl Med. 2022 Apr 2;20:151. doi: 10.1186/s12967-022-03350-6 (PMC8976335; doi:10.1186/s12967-022-03350-6)
Supplement: Supplementary file 4 — Additional file 4: Figure S2. Killing of NK-92 cells by either resting or activated primary blood NK cells. The susceptibility of NK-92 cells to attack by primary peripheral blood NK cells from healthy donors was investigated using NK-92 cells that were either non-irradiated or were irradiated with 10 Gy and then cultured for 1 day. The primary NK cells were either resting or activated by 3 days culture with 1000 u/ml IL-2. Cytotoxicity was measured using 4-h 51Cr-release assays; the horizontal gray dashed line reflects the 10% threshold for positive killing. The results illustrated represent assays of multiple donors across multiple experiments. [file 12967_2022_3350_MOESM4_ESM.pdf]

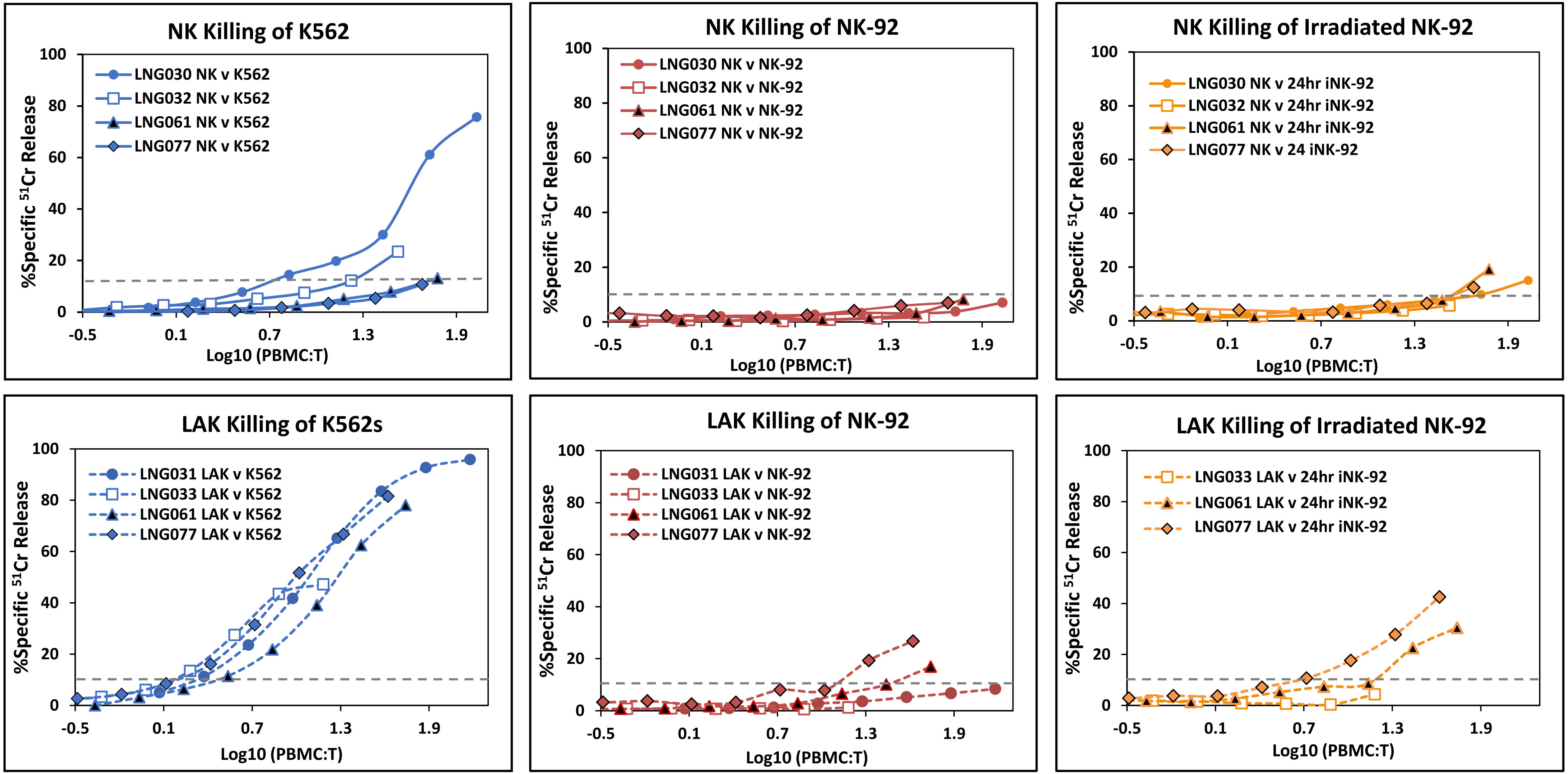

Figure S2. Killing of NK-92 cells by either resting or activated primary blood NK cells. The susceptibility of NK-92 cells to attack by primary peripheral blood NK cells from healthy donors was investigated using NK-92 cells that were either non-irradiated or were irradiated with 10 Gy and then cultured for one day. The primary NK cells were either resting or activated by three days culture with 1000 u/ml IL-2. Cytotoxicity was measured using 4-hour ^{51}Cr-release assays; the horizontal gray dashed line reflects the 10% threshold for positive killing. The results illustrated represent assays of multiple donors across multiple experiments. LNG identifier corresponds to experiment number.
